# Toll-Like Receptor 2 Stimulation of Osteoblasts Mediates *Staphylococcus Aureus* Induced Bone Resorption and Osteoclastogenesis through Enhanced RANKL

**DOI:** 10.1371/journal.pone.0156708

**Published:** 2016-06-16

**Authors:** Ali Kassem, Catharina Lindholm, Ulf H Lerner

**Affiliations:** 1 Department of Molecular Periodontology, Umeå University, Umeå, Sweden; 2 Centre for Bone and Arthritis Research, Department of Internal Medicine and Clinical Nutrition at Institute for Medicine, Sahlgrenska Academy at University of Gothenburg, Gothenburg, Sweden; 3 Department of Rheumatology and Inflammation Research, Institute of Medicine, Sahlgrenska Academy, University of Gothenburg, Gothenburg, Sweden; China Medical University, TAIWAN

## Abstract

Severe *Staphylococcus aureus* (*S*. *aureus*) infections pose an immense threat to population health and constitute a great burden for the health care worldwide. *Inter alia*, *S*. *aureus* septic arthritis is a disease with high mortality and morbidity caused by destruction of the infected joints and systemic bone loss, osteoporosis. Toll-Like receptors (TLRs) are innate immune cell receptors recognizing a variety of microbial molecules and structures. *S*. *aureus* recognition via TLR2 initiates a signaling cascade resulting in production of various cytokines, but the mechanisms by which *S*. *aureus* causes rapid and excessive bone loss are still unclear. We, therefore, investigated how *S*. *aureus* regulates periosteal/endosteal osteoclast formation and bone resorption. *S*. *aureus* stimulation of neonatal mouse parietal bone induced *ex vivo* bone resorption and osteoclastic gene expression. This effect was associated with increased mRNA and protein expression of receptor activator of NF-kB ligand (RANKL) without significant change in osteoprotegerin (OPG) expression. Bone resorption induced by *S*. *aureus* was abolished by OPG. *S*. *aureus* increased the expression of osteoclastogenic cytokines and prostaglandins in the parietal bones but the stimulatory effect of *S*. *aureus* on bone resorption and *Tnfsf11* mRNA expression was independent of these cytokines and prostaglandins. Stimulation of isolated periosteal osteoblasts with *S*. *aureus* also resulted in increased expression of *Tnfsf11* mRNA, an effect lost in osteoblasts from *Tlr2* knockout mice. *S*. *aureus* stimulated osteoclastogenesis in isolated periosteal cells without affecting RANKL-stimulated resorption. In contrast, *S*. *aureus* inhibited RANKL-induced osteoclast formation in bone marrow macrophages. These data show that *S*. *aureus* enhances bone resorption and periosteal osteoclast formation by increasing osteoblast RANKL production through TLR2. Our study indicates the importance of using different *in vitro* approaches for studies of how *S*. *aureus* regulates osteoclastogenesis to obtain better understanding of the complex mechanisms of *S*. *aureus* induced bone destruction *in vivo*.

## Introduction

Severe *Staphylococcus aureus* (*S*. *aureus*) infections are a huge burden to healthcare systems worldwide. *S*. *aureus* causes a wide range of infectious diseases, from minor skin infections to life-threatening infections like endocarditis, toxic shock syndrome or sepsis. It can also cause post-operative wound- and implant-infections [1]. The emergence of multi-resistant *S*. *aureus* strains, Methicillin-resistant *S*. *aureus* (MRSA), causing infections that are difficult to treat makes the healthcare burden even more complicated [2]. *S*. *aureus* is a commensal bacterium, colonizing approximately 30% of the adult population [3] that can be highly opportunistic and invasive due to several virulence factors such as cell surface proteins and toxins. These virulence factors give *S*. *aureus* the ability to evade and destroy the host immune system and many of the clinical symptoms seen in patients are correlated with these virulence factors [4].

Osteomyelitis is a global, serious and morbid condition, especially in children, affecting bone tissue due to *S*. *aureus* infection in bone marrow. Acute osteomyelitis is characterized by rapid necrosis and destruction in bone and suppuration, while chronic osteomyelitis is often associated with sclerosing periosteal bone formation [5–8].

Arthritis can be a consequence of certain bacterial infections of which *S*. *aureus* is the most common pathogen in adults and children [9–11]. Infectious or septic arthritis is a rapid and progressive condition with high morbidity, characterized by joint swelling and early destruction of joint cartilage and bone, but also by systemic bone loss [12,13]. Septic arthritis has a prevalence of up to 0.01% in the general population and is seven times higher in patients with rheumatoid arthritis and prosthetic joints [5,14]. Bone loss in experimentally induced *S*. *aureus* septic arthritis in mice can be inhibited by treatment with bisphosphonate, OPG-Fc or RANK-Fc, demonstrating the importance of excessive osteoclast formation as a cause of bone loss [15,16]. Systemic bone loss is partly mediated by *S*. *aureus* lipoprotein since a lipoprotein-deficient mutant strain causes less bone loss [17].

Increased orthopedic implant failure facilitated by *S*. *aureus* infections constitutes a vast and costly issue for the health care system and the society [18]. *S*. *aureus* is also present abundantly in clinical sites of periodontitis and peri-implantitis [19]. Locally applied *S*. *aureus* in the gingiva causes osteoclast formation in alveolar bone and periodontal bone loss and, therefore, suggested being able to induce and synergistically enhance periodontal destruction [20,21].

Osteoclasts are multinucleated giant cells generated by fusion of hematopoietic mononuclear osteoprogenitor cells from the myeloid origin [22]. The differentiation of these progenitor cells requires activation of the receptor c-Fms (colony stimulating factor 1 receptor, CSF1R) by its ligand macrophage colony-stimulating factor (M-CSF or colony stimulating factor 1/CSF1), which stimulates proliferation and survival of the progenitors. Subsequent activation of the receptor activator of NF-κB (RANK) with RANKL (RANK-Ligand), expressed by osteoblasts/osteocytes, induces differentiation along the osteoclastic lineage [23,24]. Interaction between RANKL and RANK can be inhibited by the decoy receptor osteoprotegerin (OPG), which binds and neutralizes RANKL. Osteoclasts resorb bone by initially dissolving the hydroxyapatite crystals in bone matrix through release of protons. Subsequently, degradation of organic matrix (mainly type I collagen) by various proteolytic enzymes will follow. One important bone matrix degrading enzyme is cathepsin K [25]. Osteoblasts, from mesenchymal origin, are the cells responsible for bone formation by producing bone matrix proteins and then depositing mineral crystals in the matrix. Osteoblasts/osteocytes also are key cells for the control of bone resorption by expressing and secreting RANKL [26,27].

Several studies have shown that *S*. *aureus* can be recognized by osteoblasts affecting their bone forming activities as well as their effects on osteoclastogenesis. It has been shown that *S*. *aureus* can inhibit bone formation and expression of bone formation genes *in vitro* in human primary osteoblasts and osteoblastic cell line MG63 [28,29]. In the mouse osteoblastic cell line MC3T3-E1, *S*. *aureus* similarly decreases osteogenic differentiation and induces apoptosis [30]. *S*. *aureus* also upregulates RANKL mRNA and protein in primary mouse and human osteoblasts and in the mouse osteoblastic cell line MC3T3-E1 [17,28,31,32]. Interestingly, *S*. *aureus* protein A binds to tumor necrosis factor recptor-1 on osteoblasts causing decreased expression of bone formation genes and increased expression of inflammatory cytokines such as interleukin-6 (IL-6) [32,33]. It has also been reported that *S*. *aureus* can upregulate the expression of death inducing receptors (DR4 and DR5) leading to osteoblast apoptosis and increased OPG release [34]. These *in vitro* observations suggest that increased osteoclast formation caused by *S*. *aureus* may be due to *S*. *aureus* primarily targeting osteoblasts, which respond with increased RANKL expression.

In addition to the studies showing that *S*. *aureus* can interact with osteoblasts, it has been shown that this bacterium can be recognized by osteoclast progenitors. Using either live *S*. *aureus*, or *S*. *aureus* cell wall peptidoglycan or lipoteichoic acid, it has been found that all these preparations inhibit RANKL induced osteoclast formation in mouse bone marrow macrophage cultures [21,35,36], while stimulating differentiation along the macrophage lineage [36]. When using RANKL primed bone marrow macrophages, however, *S*. *aureus* cell wall peptidoglycan stimulated osteoclast formation [21]. In crude bone marrow cell cultures, containing both stromal cells/osteoblasts and hematopoietic cells, addition of surface-associated material from *S*. *aureus* enhanced osteoclastogenesis [37].

Due to the severity of *S*. *aureus* septic arthritis and to the increased use of prosthetic joint replacement with a risk of *S*. *aureus* infections, it is important to understand the bone destructive mechanisms exerted by *S*. *aureus* in order to develop new treatment strategies. Studies on effects by *S*. *aureus* on osteoclast formation have been performed using osteoclast progenitors from bone marrow showing either inhibition or stimulation of osteoclast formation. However, mature osteoclasts are formed exclusively on periosteal and endosteal surfaces and we, therefore, have studied how *S*. *aureus* can regulate osteoclastogenesis in the periosteum/endosteum. For this purpose we have used either *ex vivo* organ cultures of mouse parietal bones, or cell cultures containing periosteal/endosteal osteoblasts and osteoclast progenitors. We found that bone resorption and osteoclast formation caused by stimulation of organ cultured parietal bones or periosteal/endosteal cell cultures with RANKL was not affected by *S*. *aureus*. In contrast, *S*. *aureus* abolished RANKL induced osteoclastogenesis in bone marrow macrophage cultures. These observations demonstrate that regulation of osteoclastogenesis is different using osteoclast progenitor cells from different tissues. Most importantly, *S*. *aureus* stimulated bone resorption and osteoclast formation in both organ cultured bone and periosteal/endosteal cell cultures, similar to *in vivo* observations in humans and rodents with *S*. *aureus* infections, and this response was dependent on TLR2-mediated increase of RANKL.

## Material and Methods

### Bacteria

The two *S*. *aureus* isolates, one Toxic shock syndrome toxin 1 (TSST-1) and Staphylococcal Enterotoxin A (SEA) producing, and one non-toxin producing strain, used in this study were originally isolated from healthy Swedish infants as previously described [38]. After 24 h growth on horse blood agar plates, harvested bacteria were washed in phosphate buffered saline (PBS), inactivated by exposure to UV-light (280–315 nm), and suspended in sterile PBS before use. Complete UV inactivation was confirmed by control cultures. Bacterial preparations were stored at—70°C until use.

### Mice

CsA mice from our inbred colony, CB57BL/6J and B6.129 Tlr2^tm1Kir^/J mice were from Jackson Laboratories. The mice were maintained (≤10 in each cage) under standard conditions of temperature and light, and were fed with standard laboratory chow and water ad libitum. Adult mice were killed by cervical dislocation and newborn mice by decapitation. 298 adult and newborn mice were used for this study. The Ethical committee of Umeå University, Umeå, Sweden has approved the animal care and experiments.

### Reagents

Essentially fatty acid-free bovine serum albumin (BSA), tartrate-resistant acid phosphatase (TRAP) staining-kit (Sigma-Aldrich); alpha minimum essential medium (α-MEM), zoledronic acid, and indomethacin (Invitrogen); [^45^Ca]CaCl_2_ (Amersham Biosciences); oligonucleotide primers and probes, L-glutamine (Invitrogen or Applied Biosystems); TLR2 agonist (Palmitoyl-2-Cys-Ser-(Lys)_4_) Pam2, lipoprotein-containing lipopolysaccharide from *Porphyromonas gingivalis* (LPS *P*. *gingivalis*), heat killed *Listeria Monocytogenes* (HKLM) (InvivoGen); antibiotics (AstraZeneca); culture dishes, multiwell plates (Nunc Inc.); mouse recombinant OPG, RANKL, M-CSF, IL-1β, IL-6, IL-6sR, IL-11, oncostatin (OSM), leukemia inhibitory factor (LIF), tumor necrosis factor-α (TNF-α), anti-IL-1β (MAB401), anti-IL-6 (MAP406), anti-IL-11 (AF 418 NA), anti-OSM (AF 495 NA), anti-LIF (AF 449), anti-TNF-α (MAB 4101) (R&D Systems); RatLaps^™^ CTX ELISA kit (Immonodiagnosticsystems); Prostaglandin E_2_ [^125^I]-RIA^®^ Kit (Perkin-Elmer); RNAqueous–4 PCR^®^ kit (Ambion); High-Capacity cDNA Reverse Transcription^®^ Kit (Applied Biosystems); Kapa2G^™^ Robust HotStart PCR kit, Kapa^™^ Probe Fast qPCR kit (KapaBiosystems). Bacteria, antibodies and all other test substances, with the exception of indomethacin, were dissolved in culture media. Indomethacin was dissolved in ethanol; the final concentration of ethanol did not exceed 0.1%, a concentration which we have previously found not to affect bone resorption in the parietal bone cultures.

### Organ culture of mouse parietal bones

Parietal bones from 5–7 days-old mice were dissected and cut either into halves for most of the experiments, or into quarters for mineral release analyses. Subsequently, the bones were incubated for 24 h in serum free α-MEM containing BSA (0.1%) and indomethacin (1 μM) to prevent the initial effect of released prostaglandins due to the dissection trauma [39,40]. The bones were then washed extensively with sterile PBS and cultured in indomethacin free media with or without *S*. *aureus* or other test substances.

### Bone resorption assays

Bone resorption was analyzed by assessing either release of mineral (^45^Ca) or of matrix degradation fragment (CTX) from the bones to the culture media. 2–3 days-old mice were injected with 1.5 μCi ^45^Ca 4–5 days prior to dissection, and the amounts of radioactivity in bone and culture medium were analyzed by liquid scintillation at the end of the culture period. For the time-course experiments, the mice were injected with 12.5 μCi ^45^Ca, and the radioactivity was analyzed at different time points by withdrawal of small amounts of the culture media. Isotope release was expressed as the percent release of the initial amount of isotope (calculated as the sum of radioactivity in medium and bone after culture).

The release of collagen fragments (CTX) from the bone matrix into the media was analyzed by RatLaps^™^ ELISA kit.

### Isolation and culture of parietal cells

Periosteal and endosteal cells were isolated from 2–3 days-old mouse parietal bones by sequential collagenase digestion [41]. Pooled cells from populations 1–10, containing both osteoblast and osteoclast progenitors capable of forming bone resorbing osteoclasts [41], were used for osteoclastogenesis experiments. These cells were initially cultured in 25 cm^2^ flasks with α-MEM containing 10% FBS for 48 h to expand the number of cells. Cells were then washed and detached and subsequently seeded in 12-multiwells (10^4^ cells/cm^2^) and cultured with or without *S*. *aureus* or other test substances for 9 days. Cells were fixed and stained for TRAP.

Cells from populations 6–10 are enriched for osteoblastic cells and widely used for osteoblastogenesis experiments. These cells were expanded as described above, and seeded in 24-multiwells (10^4^ cells/cm^2^) and incubated with or without test substances for 48 h at which time point RNA was isolated and used for gene expression analyses.

### Bone marrow macrophage isolation and cultures

Mouse bone marrow cells were isolated from tibia and femur as described [42]. The bone marrow macrophages were purified by incubating the cells on Corning dishes in the presence of M-CSF (30 ng/ml) for 48 h. The adherent bone marrow macrophages (BMM) were used as osteoclast progenitor cells. These cells do not contain T- or B-cells and all cells express the macrophage marker CD11b/Mac-1 [43]. After washing and detaching, cells were spot-seeded (5x10^3^ cells in 10 μl) at the center of 96-multiwells and left to adhere for 10 min. Then, the wells were added 200 μl medium containing M-CSF (30 ng/ml; controls) or M-CSF (30 ng/ml) +RANKL (4 ng/ml) with or without *S*. *aureus* or other test substances and incubated for 96 h. In experiments with committed osteoclast progenitors, cells were primed with RANKL (4 ng/ml) in presence of M-CSF for 24 h. Cells were then washed and medium containing M-CSF with or without test substances was added. At the end of the cultures, cells were fixed and stained for TRAP.

### TRAP staining

Cells were fixed, washed and stained for TRAP using the Naphtol AS-BI phosphate kit from Sigma Aldrich. TRAP^+^ cells with at least three nuclei were counted as TRAP^+^ multinucleated osteoclasts (TRAP^+^MuOCL).

### Gene expression analyses

RNA was isolated from bone tissue or cells using RNAqueous–4 PCR^®^ kit, according to manufacturer′s instructions. The RNA was quantified spectrophotometrically and single-stranded cDNA was synthesized from 0.1–0.5 μg of total RNA using High High-Capacity cDNA Reverse Transcription^®^ Kit. To ensure absence of genomic DNA in the samples, negative controls with no MultiScribe^™^ reverse transcriptase were included. The following predesigned real-time PCR assays from Applied Biosystems were used for gene expression assays: *Acp5* (Mm00475698_m1), *Calcr* (Mm00432282_m1), *c-Fos* (Mm00487425_m1), *Csf1* (Mm00432686_m1), *Csf1r* (Mm01266652_m1), *Ctsk* (Mm00484036_m1), *Il1b* (Mm00434228_m1), *Il11* (Mm00434162_m1), *Il6* (Mm00446190_m1), *Lif* (Mm00434761_m1), *Nfatc1* (Mm00479445_m1), *Oscar* (Mm00558665_m1), *Osm* (Mm01193966_m1), *Ptgs2* (Mm00478374_m1), *Tnfsf2* (Mm00443258_ml), *Tnfsf11* (Mm00441908_m1), *Tnfrsf11a* (Mm00437135_m1), *Tnfrsf11b* (Mm00435452_m1). *β-actin* (4352341E) was used as a reference gene to normalize for variability in amplification due to possible differences in starting mRNA concentrations. ABI PRISM 7900 HT Sequence Detection System and Software were used for the amplifications.

### RANKL and OPG protein analyses

Assessment of RANKL and OPG protein was made using ELISA kits after lysing the bones in 1 ml 0.2% Triton X-100. The sensitivities of the immunoassays are 5 pg/ml.

### Prostaglandin E_2_ analysis

The amount of released PGE_2_ in the culture media was measured by a radioimmunoassay kit.

### Neutralizing antibody experiments

Neutralizing antibodies against mouse interleukin-1β (IL-1β), IL-6, IL-11, leukemia inhibitory factor (LIF), oncostatin M (OSM) and tumor necrosis factor-α (TNF-α) were used to elucidate the role of these cytokines in *S*. *aureus* induced bone resorption. We first validated the efficacy of the antibodies by verifying that the antibodies neutralized the effect of IL-1β, IL-6+sIL-6R, IL-11, LIF, OSM or TNF-α (all used at maximally effective concentrations) on mRNA expression of *Tnfsf11* in parietal bones. The parietal bones, after indomethacin pretreatment, were preincubated with the antibodies for 8 h prior to the stimulation with *S*. *aureus*. To eliminate the possibility that several cytokines were responsible for the effects, we added the antibodies all together to the cell and organ cultures in a mixture at final concentrations of 1μg/ml for anti-IL-6, anti-IL-11, anti-LIF and anti-TNF-α, 3 μg/ml for anti-OSM and 5 μg/ml for anti-IL-1β, and analyzed the response on the expression of *Tnfsf11* mRNA or CTX release, respectively.

### Statistics

Statistical analyses were performed using Paired t-test (S3A–S3D Fig) or one-way ANOVA (all other experiments) with Shapiro-Wilk’s normality test and Holm-Sidak’s *post hoc* test using SigmaPlot software, (Systat Software Inc). The means and SEM shown in each figure are based upon 5–6 calvarial bones or cell culture wells in separate experiments, as specified in the legends to figures. All experiments were repeated with comparable results. Data were considered statistically significant when *P*< 0.05 (*), *P*<0.01 (**) and *P*<0.001 (*** or $).

## Results

### *S*. *aureus* stimulates bone resorption in parietal bones

**Fig 1:**
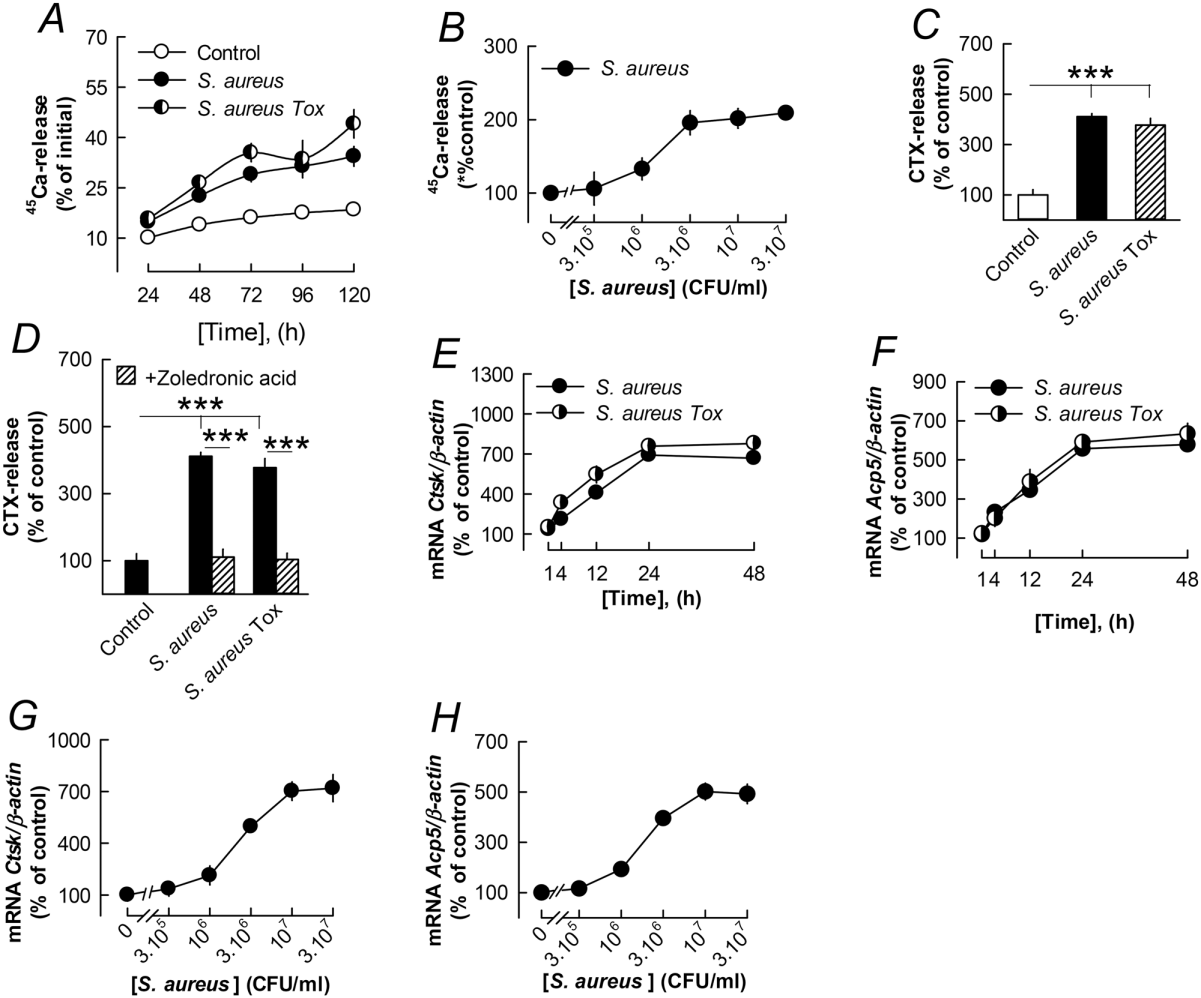
*S*. *aureus* stimulates bone resorption and expression of osteoclastic and osteoclastogenic genes in organ cultures of neonatal mouse parietal bones. (**A**-**C**) *S*. *aureus* time- and concentration-dependently increased ^45^Ca and CTX release from the parietal bones. (**D**) The stimulatory effect by *S*. *aureus* (3x10^6^ CFU/ml) on CTX release was inhibited by zoledronic acid (0.2 μmol/l). (**E**, **F**) *S*. *aureus* (3x10^6^ CFU/ml) time-dependently upregulated the mRNA expression of *Ctsk* and *Acp5* in the parietal bones. (**G**, **H**) Concentration-dependent effects by *S*. *aureus* on *Ctsk* and *Acp* mRNA expression in the parietal bones. Data are means of 6 (A-D) or 5 (E-H) observations and SEM is given as vertical bars when larger than the radius of the symbol. In Fig 1A, all effects at 48 h and later were statistically significant (*P*<0.001) with the exception of *S*. *aureus Tox* at 96 h (*P*<0.01); at 24 h effects were also significant (*P*<0.05). In Fig 1B, effects by 3x10^6^–3x10^7^ CFU/ml were statistically significant (*P*>0.01). In Fig 1G, effects were statistically significant at 10^6^ (*P*<0.01) and 3x10^6^–3x10^7^ (*P*<0.001) CFU/ml. In Fig 1H, effects by 10^6^–3x10^7^ CFU/ml were statistically significant (*P*<0.001). ***P<0.001 compared to unstimulated control (C, D) or to *S*. *aureus* stimulated bones (D).

Both the non-toxin producing *S*. *aureus* and toxin producing *S*. *aureus* (*S*. *aureus Tox*) enhanced the release of mineral (^45^Ca) from cultured neonatal parietal bones in a time-dependent manner (Fig 1A). The effect was statistically significant (*P*<0.05) already at 24 h. The stimulatory effect of *S*. *aureus* on ^45^Ca release was concentration-dependent (Fig 1B). *S*. *aureus* and *S*. *aureus Tox* also significantly enhanced the release of bone matrix degradation fragments (CTX) from these bones (Fig 1C).

The *S*. *aureus* and *S*. *aureus Tox* induced CTX release from neonatal parietal bones was abolished by the bisphosphonate zoledronic acid (Fig 1D).

Mineral release and matrix degradation induced by *S*. *aureus* and *S*. *aureus Tox* were associated with time-dependent increased mRNA expression of *Ctsk* (encoding cathepsin K; Fig 1E) and *Acp5* (encoding TRAP; Fig 1F). The increased mRNA expression of *Ctsk* and *Acp5* was dependent on the concentration of *S*. *aureus* (Fig 1G and 1H).

### *S*. *aureus*-induced osteoclast formation and bone resorption in parietal bones is mediated by enhanced RANKL

Osteoclastogenesis requires activation of c-Fms by its ligand M-CSF and activation of RANK by its ligand RANKL, with OPG being a decoy receptor for RANKL [22]. In addition, activation of the receptor OSCAR (Osteoclast-associated receptor) is important for osteoclast differentiation [44,45]. Downstream signaling includes activation of NFATC1 (Nuclear factor of activated T-cells c1) which is regarded as the master transcription factor of osteoclastogenesis [46]. We assessed the effect by *S*. *aureus* on these cytokines, receptors and transcription factor in the parietal bones.

**Fig 2:**
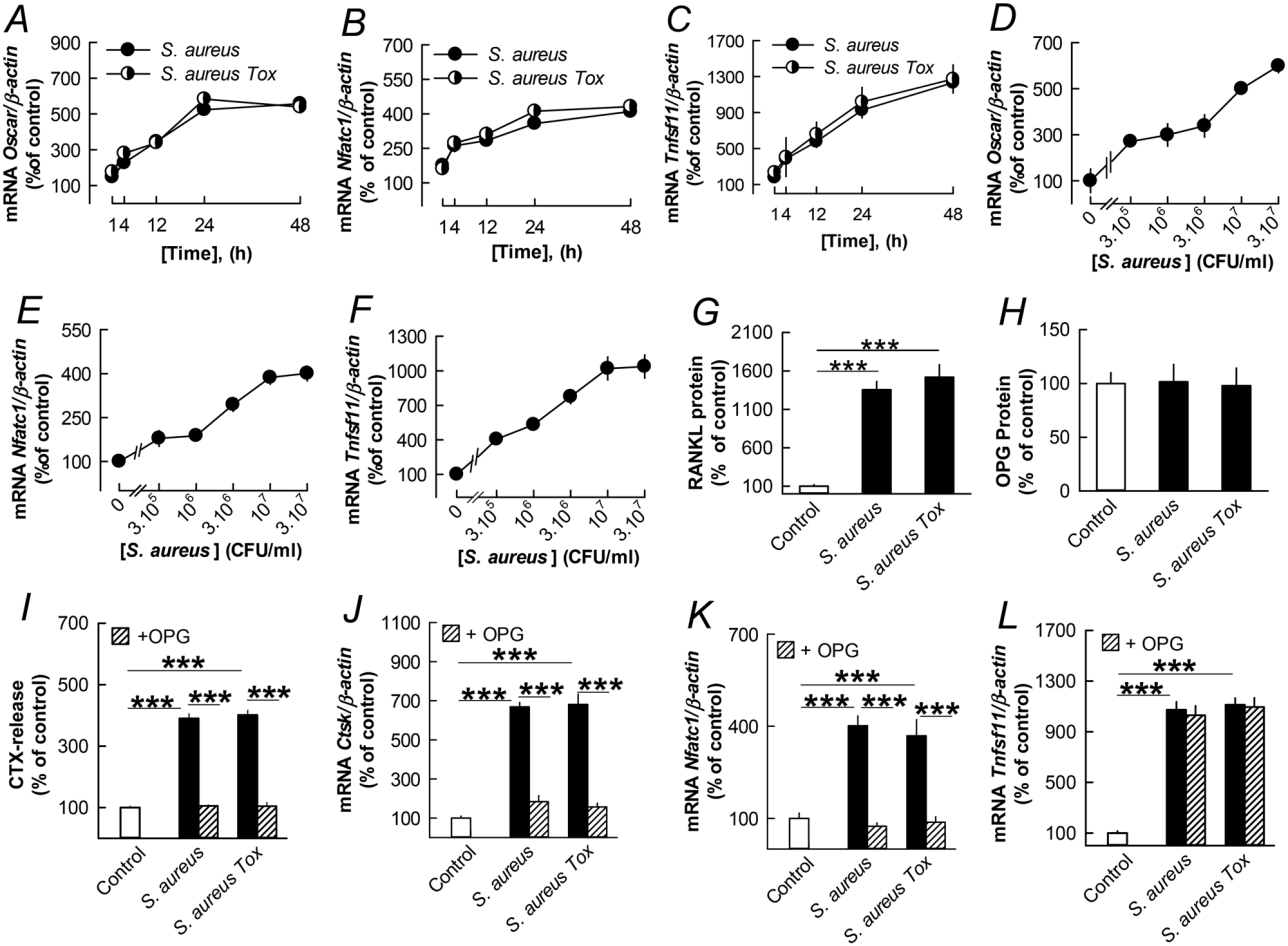
The stimulatory effect on bone resorption in mouse parietal bones by *S*. *aureus* is dependent on increased RANKL. (**A**-**C**) *S*. *aureus* (3x10^6^ CFU/ml) time-dependently increased the mRNA expression of *Oscar*, *Nfatc1* and *Tnfsf11*in the parietal bones. (**D**-**F**) Concentration-dependent effect by *S*. *aureus* on *Oscar*, *Nfatc1* and *Tnfsf11* mRNA. (**G**) *S*. *aureus* (3x10^6^ CFU/ml) increased the cellular level of RANKL protein without affecting OPG protein. (**I**-**L**) The stimulatory effect by *S*. *aureus* (3x10^6^ CFU/ml) on CTX release and mRNA expression of *Ctsk* and *Nfatc1* in the parietal bones was inhibited by OPG (300 ng/ml), without any effect of *Tnfsf11* mRNA expression. Data are means of 5 (A-F, J -L) or 6 (G-I) observations and SEM is given as vertical bars when larger than the radius of the symbol. In Fig 2A, effects were statistically significant at 4 h (*P*<0.05) and at 12–48 h (*P*<0.001). In Fig 2B, effects at 4–48 h were statistically significant (*P*<0.001). In Fig 2C, effects were statistically significant at 4 h (*P*<0.05) and at 12–48 h (*P*<0.001). In Fig 2D, effects were statistically significant at 3 x 10^5^, 10^7^ and 3 x10^7^ (*P*<0.001) and at 10^6^ and 3 x 10^6^ *(P*<0.01) CFU/ml. In Fig 2E and F effects at 3 x 10^5^–3 x 10^7^ CFU/ml were statistically significant (*P*<0.001). ***P<0.001 compared to unstimulated control (G, I-L) or to *S*. *aureus* stimulated bones (I-K).

*S*. *aureus* and *S*. *aureus Tox* time-dependently stimulated the mRNA expression of *Oscar*, *Nfatc1* and *Tnfsf11* (encoding RANKL) (Fig 2A–2C). The stimulatory effect on these transcripts was dependent on the concentration of *S*. *aureus* (Fig 2D–2F). The bacterium also increased the expression of *Csf1r* (encoding the M-CSF receptor c-Fms) and *Csf1* (encoding M-CSF) (S1A–S1D Fig), whereas *Tnfrsf11a* (encoding RANK) and *Tnfrsf11b* (encoding OPG) mRNA were unaffected (S1E–S1H Fig).

In agreement with the mRNA analyses, *S*. *aureus* and *S*. *aureus Tox* significantly enhanced RANKL protein levels in the parietal bones (Fig 2G), without significantly affecting OPG protein (Fig 2H).

The importance of increased RANKL/OPG ratio for the stimulatory effect on bone resorption in neonatal parietal bones was demonstrated by the observation that bone matrix degradation (CTX release) in parietal bones challenged by *S*. *aureus* and *S*. *aureus Tox* was abolished when recombinant OPG (300 ng/ml) was added (Fig 2I). OPG also inhibited *S*. *aureus* induced mRNA expression of *Ctsk* (Fig 2J) and *Nfatc1* (Fig 2K), indicating that OPG inhibited bone resorption through inhibition of osteoclast differentiation. The fact that OPG did not affect *Tnfsf11* mRNA (Fig 2L) shows that OPG acted downstream RANKL formation to inhibit osteoclastogenesis.

### The role of osteoclastogenic cytokines and prostaglandins in *S*. *aureus* induced RANKL and bone resorption

**Fig 3:**
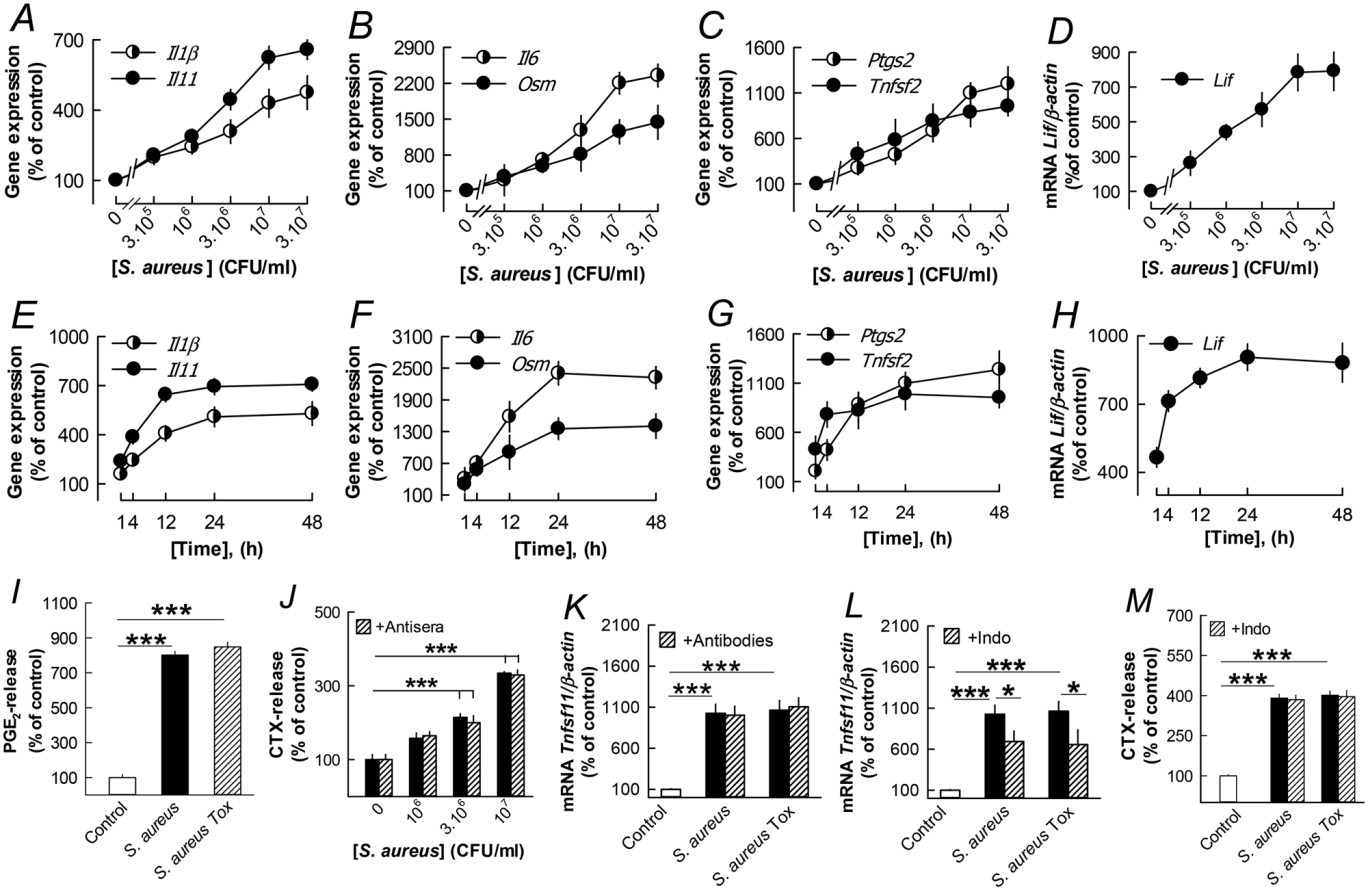
*S*. *aureus* stimulates bone resorption in mouse parietal bones independently on cytokine and prostaglandin production. (**A**-**D**) Concentration-dependent effect by *S*. *aureus* on *Il1b*, *Il11*, *Il6*, *Osm*, *Tnfsf2* and *Ptgs2* mRNA expression in parietal bones. (**E**-**H**) Time-dependent effect by *S*. *aureus* (3x10^6^ CFU/ml) on *Il1b*, *Il11*, *Il6*, *Osm*, *Tnfsf2* and *Ptgs2* mRNA expression in parietal bones. (**I**) Stimulation of PGE_2_ release from the bones by *S*. *aureus* (3x10^6^ CFU/ml). (**J**, **K**) The stimulatory effect by *S*. *aureus* (10^6^–10^7^ CFU/ml) on CTX- release and by *S*. *aureus* (3x10^6^ CFU/ml) on *Tnfsf11* mRNA expression in parietal bones was unaffected by adding a mixture of antibodies neutralizing IL-1β, IL-6, IL-11, LIF, OSM and TNF-α. (**L**, **M**) Indomethacin (1 μmol/l) partially reduced *Tnfsf11* mRNA induced by *S*. *aureus* (3x10^6^ CFU/ml) but did not affect CTX-release. Data are means of 5 (A-H, K, L) or 6 (I, J, M) observations and SEM is given as vertical bars when larger than the radius of the symbol. In Fig 3A, effects on *Il11* (*P*<0.001) and *Il1b* (*P*<0.01) mRNA were statistically significant at 3 x 10^5^–3 x 10^7^ CFU/ml. In Fig 3B, effects on *Il6* (*P*<0.001) and *Osm* (*P*<0.01) mRNA were statistically significant at 3x10^5^–3x10^7^ CFU/ml. In Fig 3C, effects on *Ptgs2* mRNA were statistically significant (*P*<0.001) at 3x10^5^–3x10^7^ CFU/ml and on *Tnfsf2* mRNA at 3x10^5^ and 3x10^7^ (*P*<0.001) and at 10^6^–10^7^ (*P*<0.01) CFU/ml. In Fig 3D, effects on *Lif* mRNA at 3x10^5^ (*P*<0.05) and at 10^6^–3x10^7^ (*P*<0.01) CFU/ml were statistically significant. In Fig 3E, effects on *Il1b* and *Il11* mRNA were statistically significant (*P*<0.001) at 4–48 h. In Fig 3F, effects on *Il6* mRNA were statistically significant (*P*<0.001) at 1–48 h and on *Osm* mRNA at 1, 4, 24 and 48 h (*P*<0.001) and at 12 h (*P*<0.01). In Fig 1G, effects were statistically significant on *Ptgs2* mRNA at 4–48 h (*P*<0.001) and on *Tnfsf2* mRNA at 4, 24 and 48 h (*P*<0.001) and at 12 h (*P*<0.01). In Fig 3H, effects on *Lif* mRNA were statistically significant (*P*<0.001) at 1–48 h. ***P<0.001 compared to unstimulated control (I-M) or *P<0.05 to *S*. *aureus* stimulated bones (L).

*S*. *aureus* enhanced the mRNA expression of *Il1b*, *Il6*, *Il11*, *Lif*, *Osm*, *Tnfsf2* (encoding TNF-α) and *Ptgs2* (encoding cyclooxygenase-2) in the parietal bones in a dose- and time-dependent manner (Fig 3A–3H), as expected since TLR2 activation often results in enhanced expression of these proinflammatory molecules [47]. *S*. *aureus* as well as *S*. *aureus Tox* enhanced the release of PGE_2_ from the parietal bones (Fig 3I).

Since these cytokines and prostaglandins are osteoclastogenic and can promote bone resorption, we examined their possible role in the *S*. *aureus* induced bone resorption and enhanced *Tnfsf11* mRNA expression by using specific antibodies neutralizing the cytokines and indomethacin to inhibit prostaglandin biosynthesis. We first confirmed the efficacy of the antibodies in the organ culture assay of parietal bones (S2A–S2C Fig), and then added a mixture of antibodies neutralizing IL-1β, IL-6, IL-11, LIF, OSM and TNF-α to *S*. *aureus* stimulated bones. Addition of antibodies did not affect bone resorption induced by the bacteria at optimal or suboptimal concentrations, as assessed by bone matrix degradation (CTX release; Fig 3J). Nor did the antibodies affect the increased *Tnfsf11* mRNA expression induced by *S*. *aureus* (Fig 3K). Despite the partial decrease of *S*. *aureus* induced *Tnfsf11* mRNA expression (Fig 3L) in parietal bones by indomethacin, bone resorption was not affected (Fig 3M).

### *S*. *aureus* stimulates *Tnfsf11* in mouse parietal osteoblasts independent of cytokine induction but dependent on TLR2

**Fig 4:**
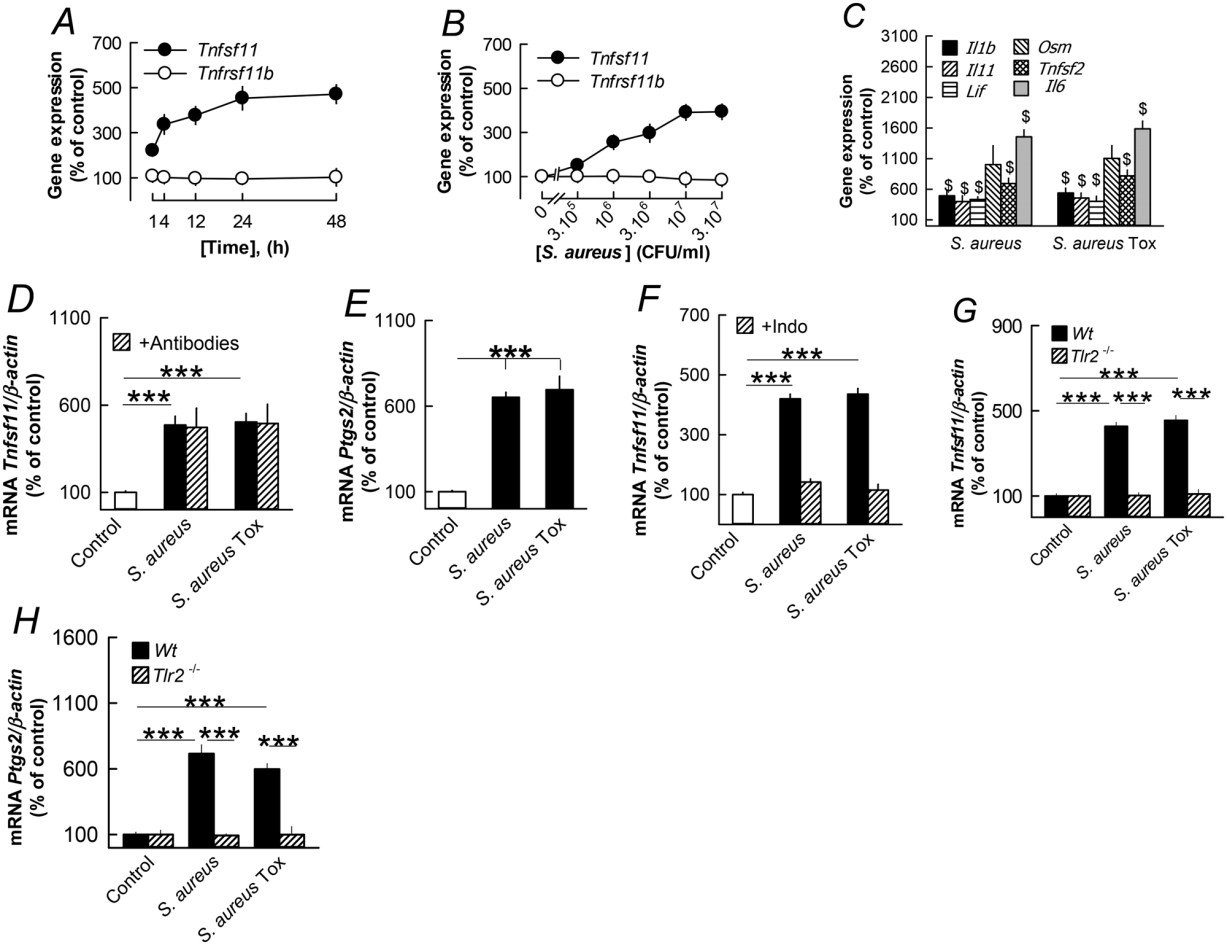
*S*. *aureus* stimulates RANKL in isolated mouse calvarial osteoblasts independent on cytokine but dependent on prostaglandin productions and TLR2. (**A**) *S*. *aureus* time-dependently stimulated *Tnfsf11* mRNA without affecting *Tnfrsf11b* mRNA in mouse osteoblasts. (**B**) Concentration-dependent stimulation of *Tnfsf11* mRNA, with no effect on *Tnfrsf11b* mRNA, by *S*. *aureus* (3x10^6^ CFU/ml). (**C**) *S*. *aureus* (3x10^6^ CFU/ml) upregulated the mRNA expression of *Il1b*, *Il11*, *Il6*, *Lif*, *Osm* and *Tnfsf2* in osteoblasts. (**D**) The stimulatory effect by *S*. *aureus* (3x10^6^ CFU/ml) on *Tnfsf11* mRNA expression in osteoblasts was unaffected by adding a mixture of antibodies neutralizing IL-1β, IL-6, IL-11, LIF, OSM and TNF-α. (**E**) *S*. *aureus* (3x10^6^ CFU/ml) stimulated *Ptgs2* mRNA in mouse osteoblasts. (**F**) Indomethacin (1 μmol/l) abolished *Tnfsf11* mRNA in osteoblasts induced by *S*. *aureus* (3x10^6^ CFU/ml). (**G, H**) The stimulatory effect by *S*. *aureus* (3x10^6^ CFU/ml) on *Tnfsf11* and *Ptgs2* mRNA was observed in osteoblasts from *wild type* mice but not from *Tlr2* deficient mice. Data are means of 5 observations and SEM is given as vertical bars when larger than the radius of the symbol. In Fig 4A, effects on *Tnfsf11* mRNA were statistically significant at 1–48 h (*P*<0.001). In Fig 4B, effects on *Tnfsf11* mRNA were statistically significant at 10^6^ and 3x10^6^ (*P*<0.01) and at 10^7^ and 3x10^7^ (*P*<0.001) CFU/ml. ***P<0.001 compared to unstimulated control (D-G) or to *S*. *aureus* stimulated osteoblasts (G).

Osteoblasts are resident cells that communicate and activate osteoclastogenesis in bone tissue by producing RANKL in response to a variety of bone resorbing hormones and cytokines [48,49]. We, therefore, investigated if *S*. *aureus* could induce production of RANKL in mouse parietal osteoblasts. Stimulation of these cells by *S*. *aureus* caused a time- and concentration-dependent increase of *Tnfsf11* mRNA expression but had no effect on *Tnfrsf11b* mRNA expression (Fig 4A and 4B).

*S*. *aureus* and *S*. *aureus Tox* increased the mRNA expression of *Il1b*, *Il6*, *Il11*, *Lif*, *Osm and Tnfsf2* in the parietal osteoblasts (Fig 4C). Neutralization of these cytokines by a mixture of antibodies neutralizing IL-1β, IL-6, IL-11, LIF, OSM and TNF-α, did not affect the *S*. *aureus* induced *Tnfsf11* mRNA expression (Fig 4D). *S*. *aureus* and *S*. *aureus Tox* also enhanced the expression of *Ptgs2* mRNA (Fig 4E). Inhibition of prostaglandin biosynthesis by indomethacin abolished S. *aureus* and *S*. *aureus Tox* induced mRNA expression of *Tnfsf11* (Fig 4F).

Others and we [50–52] have previously shown that osteoblasts express TLR2 and we, therefore, assessed if *S*. *aureus* induced *Tnfsf11* expression was due to stimulation of TLR2. Using osteoblasts isolated from *Tlr2* deficient mice, we found that *Tnfsf11* mRNA induced by *S*. *aureus* was entirely dependent on *Tlr2* expression (Fig 4G). Similarly, *S*. *aureus* did not upregulate *Ptgs2* mRNA in osteoblasts in cells isolated from *Tlr2* deficient mice (Fig 4H).

### *S*. *aureus* differentially regulates osteoclast formation in bone marrow and periosteal cell cultures

**Fig 5:**
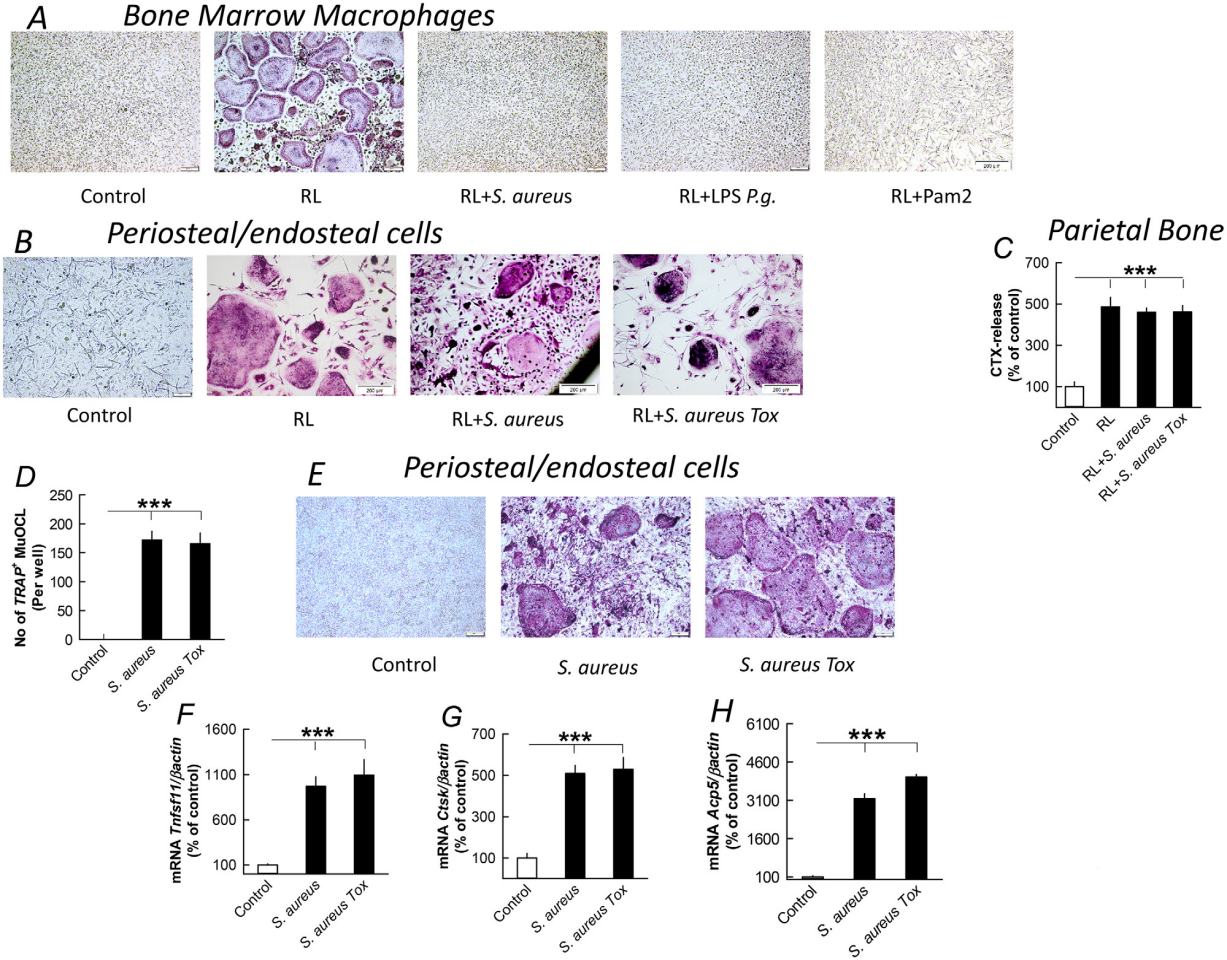
*S*. *aureus* inhibits RANKL-stimulated osteoclast formation in bone marrow macrophage cultures without affecting RANKL-stimulated osteoclast formation in periosteal/endosteal cell cultures or bone resorption in parietal bones but enhances osteoclast formation in periosteal/endosteal cell cultures in the absence of RANKL. (**A**) *S*. *aureus* (10^7^ CFU/ml), LPS *P*. *gingivalis* (*P*.*g*.; 10 μg/ml) and Pam2 (100 ng/ml) inhibited formation of TRAP^+^MuOCL in RANKL (RL; 4 ng/ml) stimulated bone marrow macrophages. (**B**) *S*. *aureus* (10^7^ CFU/ml) did not inhibit formation of TRAP^+^MuOCL in RANKL (RL; 10 ng/ml) stimulated periosteal/endosteal cell cultures. (**C**) *S*. *aureus* (10^7^ CFU/ml) did not affect RANKL (RL; 10 ng/ml) stimulated release of CTX from periosteal bone organ cultures. (**D**, **E**) *S*. *aureus* (10^7^ CFU/ml), in the absence of RANKL, stimulated formation of TRAP^+^MuOCL in periosteal/endosteal cell cultures. (**F**-**H**) *S*. *aureus* (10^7^ CFU/ml) stimulated the mRNA expression of *Tnfsf11*, *Ctsk* and *Acp5* mRNA in periosteal/endosteal cell cultures. Data are means of 6 observations and SEM is given as vertical bars. ***P<0.001 compared to unstimulated control.

Activation of TLR2 in RANKL stimulated BMM results in inhibition of osteoclast differentiation and formation [53,54]. We, therefore, asked ourselves why not *S*. *aureus* inhibited osteoclast formation in the *ex vivo* bone organ cultures. For this purpose, we compared the effects by *S*. *aureus* in three different osteoclastogenic systems, all stimulated by RANKL with or without *S*. *aureus*. *S*. *aureus*, similar to other TLR2 agonists (LPS *P*. *gingivalis* and Pam2), abolished osteoclastogenesis in RANKL stimulated BMM (Fig 5A). In contrast, *S*. *aureus* did not inhibit osteoclastogenesis induced by RANKL in periosteal/endosteal cell cultures (Fig 5B), or RANKL stimulated bone matrix degradation in parietal bone organ cultures (Fig 5C). These observations indicate that osteoclast progenitors in the periosteum/endosteum are different from those in bone marrow.

Interestingly, *S*. *aureus* stimulated osteoclast formation in the periosteal cells in the absence of exogenous RANKL (Fig 5D and 5E). The effect was associated with enhanced mRNA expression of *Tnfsf11*, *Ctsk* and *Acp5* as markers of osteoclast differentiation (Fig 5F–5H).

**Fig 6:**
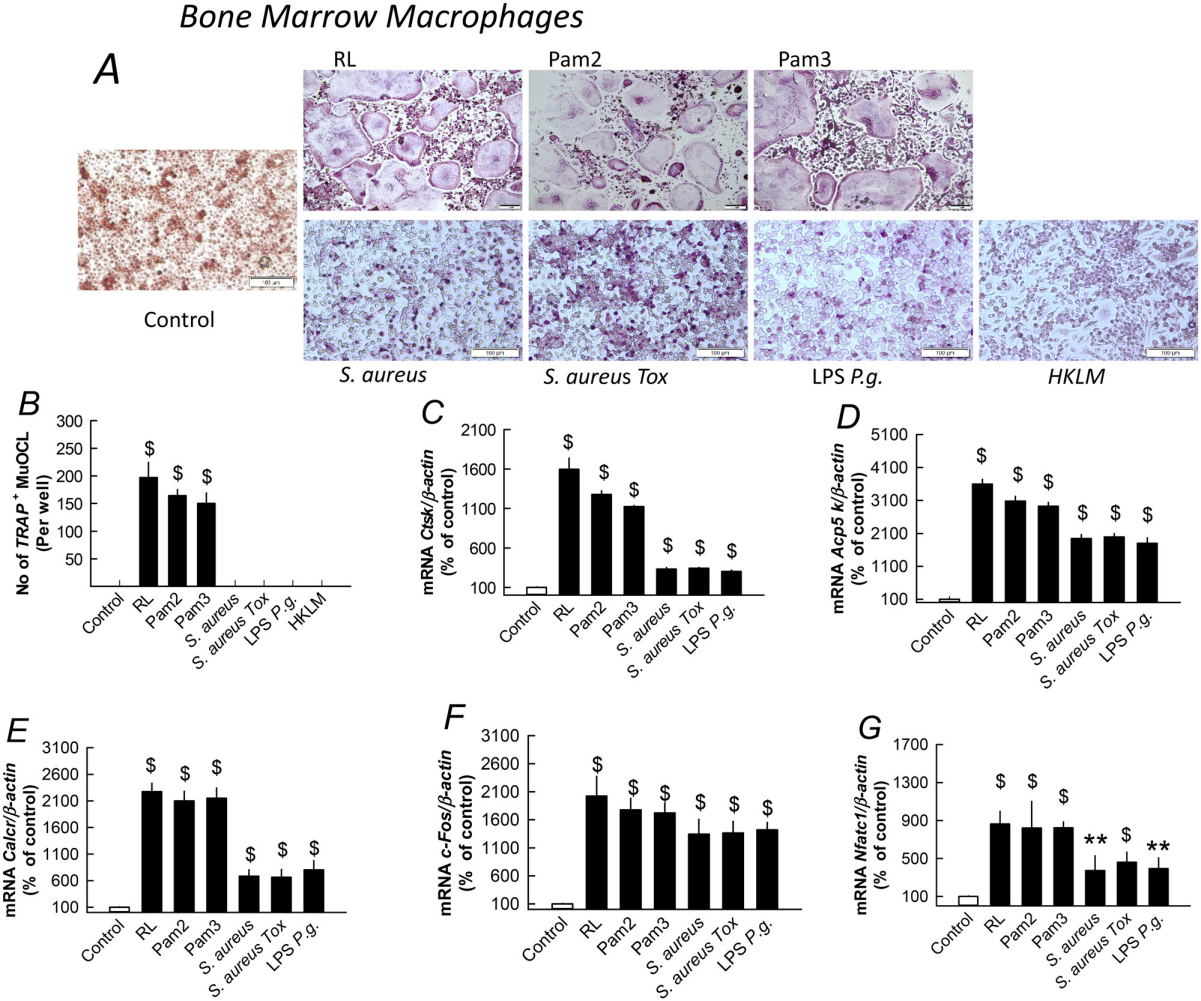
Pam 2 and Pam3 stimulate mature osteoclast formation, whereas *S*. *aureus*, LPS *P*. *gingivalis* and HKLM stimulate differentiation of mononuclear osteoclasts in RANKL-primed bone marrow macrophage cultures. Bone marrow macrophages were primed for 24 h in either M-CSF (30 ng/ml) or in M-CSF with RANKL (4 ng/ml). Then, cells were treated with M-CSF (controls) or with M-CSF and either RANKL (RL; 4 ng/ml), Pam2 (100 ng/ml), Pam3 (100 ng/ml), *S*. *aureus* (10^7^ CFU/ml), *S*. *aureus Tox* (10^7^ CFU/ml), LPS *P*. *gingivalis* (*P*.*g*.; 10 μg/ml) or HKLM (3x10^7^ UFC) for 96 h. (**A, B**) RL, Pam2 and Pam3 stimulated formation of TRAP^+^ multinucleated osteoclasts, whereas *S*. *aureus*, LPS *P*. *gingivalis* and HKLM stimulated TRAP^+^ mononucleated osteoclasts. (**C**-**D**) Effects by RL, Pam2, Pam3, *S*. *aureus*, *S*. *aureus Tox*, LPS *P*. *gingivalis* and HKLM on mRNA expression of *Ctsk*, *Acp5*, *Calcr*, *c-Fos* and *Nfatc1* in RANKL-primed bone marrow macrophages. Data are means of 6 observations and SEM is given as vertical bars. $*P*>0.001 and **P<0.01 compared to unstimulated control.

Stimulation of TLR2 by its synthetic ligand (Pam2) in committed osteoclast progenitors (RANKL-primed for 24h) has been reported to promote osteoclastogenesis [17,54]. This finding suggests the possibility that one reason for the different response to *S*. *aureus* in osteoclast progenitors from periosteum and bone marrow co-treated with RANKL might be that the differentiation stage of osteoclast progenitors determines the response to *S*. *aureus*. We, therefore, assessed the effect by *S*. *aureus* in RANKL-primed BMM. In agreement with the previous observations [17, 54], we found that Pam2 and Pam3 enhanced osteoclast formation in RANKL-primed BMM to the same degree as treatment with RANKL (Fig 6A and 6B). Unlike the synthetic ligands Pam2 and Pam3, but similar to other bacterial TLR2 ligands such as HKLM and LPS *P*. *gingivalis*, stimulation of RANKL-primed BMM with *S*. *aureus* resulted in formation of mainly mononuclear TRAP^+^ cells with only some few osteoclast-like cells (Fig 5A and 5B). This observation indicated that *S*. *aureus* can stimulate differentiation of RANKL-primed osteoclast progenitors but not to the same degree as Pam2 and Pam3 and not to the level where the progenitors fuse to typical multinucleated mature osteoclasts. Further evidence for the view that *S*. *aureus* can induce osteoclast progenitor cell differentiation were the observations that the mRNA expression of *Ctsk*, *Acp5* and *Calcr* was significantly induced by *S*. *aureus* (Fig 6C–6E), which was also true for the mRNA expression of the two osteoclastogenic transcription factors *c-Fos* and *Nfatc1* (Fig 6F and 6G). However, the degree of upregulation of these genes induced by *S*. *aureus* was clearly less than that caused by Pam2, Pam3 and RANKL. Similarly, LPS *P*. *gingivalis* induced all these osteoclast genes but the degree of stimulation was less than that induced by the compounds stimulating mature osteoclast formation (Fig 6C–6G).

### *S*. *aureus* inhibits the expression of osteoblast anabolic genes

Since *S*. *aureus* induced bone loss may not entirely depend on increased bone resorption we also assessed if the bacteria affected bone formation in the parietal bones. The mRNA expression of the bone matrix proteins osteocalcin (encoded by *Bglap*) and procollagen type I (encoded by *procol1a1*), as well as of the enzyme alkaline phosphatase (encoded by *Akp1*) was substantially decreased by *S*. *aureus* in organ cultured parietal bones (S3A–S3C Fig). This might be due to the decreased mRNA expression of the transcription factor *Runx*2 also observed (S3D Fig). Since Nfatc1 is expressed not only in osteoclasts but also in osteoblasts [55–58], and since *S*. *aureus* increased *Nfatc1* mRNA in the parietal bones (Fig 2K), we assessed if increased Nfatc1 was involved in the decreased expression of osteoblast anabolic genes. We found, however, that inhibition of these genes induced by *S*. *aureus* was independent on Nfatc1 since the inhibition of *Nfatc1* mRNA expression caused by OPG (Fig 2K) did not affect *S*. *aureus* induced down regulation of *Bglap*, *Procol1a1*, *Akp1* or *Runx2* mRNA expression (S3E–S3H Fig).

## Discussion

It is well recognized that *S*. *aureus* infections can cause local and systemic bone destruction [15–18] but the mechanisms by which *S*. *aureus* induces bone resorption are still not fully understood. Although several reports have shown that *S*. *aureus* can target osteoblasts *in vitro* causing apoptosis, decreased bone formation and decreased expression of osteoblastic genes, as well as enhanced RANKL expression [17,28,31,32], the data regarding effects on osteoclasts are more diverse. *S*. *aureus* has been shown both to inhibit [21,35,36] and stimulate [21] osteoclastogenesis in mouse bone marrow macrophage cultures depending on if the bacterium is exposed to the cells simultaneously with RANKL or after RANKL pretreatment, respectively. Since mature osteoclasts are formed only at bone surfaces we have studied the effect of *S*. *aureus* on osteoclast formation and bone resorption using osteoclast progenitors present at periosteal/endosteal surfaces.

To mimic the microenvironment of bone tissue where osteoclast formation and bone resorption take place *in vivo* we used *ex vivo* organ cultures of mouse parietal bones, exhibiting a periosteum and a thin endosteum. We show that *S*. *aureus* enhances bone resorption in the parietal bones through a process inhibited by bisphosphonate, demonstrating the importance of osteoclasts. The finding that *S*. *aureus* increased osteoclastic genes such as those encoding TRAP and cathepsin K, and the osteoclastogenic transcription factor NFATc1, showed that *S*. *aureus* induced bone resorption is due to enhanced differentiation and activation of osteoclasts.

Since the RANKL/OPG ratio is crucial for osteoclastogenesis and bone homeostasis [59,60], we next investigated the effect of *S*. *aureus* on RANKL/OPG ratio. *S*. *aureus* enhanced this ratio by increasing the expression at both mRNA and protein levels of RANKL, without affecting those of OPG in the parietal bones. The inhibition of bone resorption and osteoclastic gene expression, caused by exogenous OPG added to *S*. *aureus* stimulated bone organ cultures, further supports the essential role of RANKL in bone resorption due to *S*. *aureus* infection. Using osteoblasts from *wild type* and *Tlr2* deficient mice, we show that osteoblastic TLR2 is the receptor utilized by *S*. *aureus* in the bones to enhance RANKL. These data show that *S*. *aureus* stimulates periosteal/endosteal osteoclast formation and bone resorption in organ-cultured bones by enhancing RANKL/OPG in osteoblasts. Our observations further indicate that *S*. *aureus* does not inhibit osteoclast progenitors in these bones, in contrast to observations in bone marrow cell cultures. We cannot exclude, however, that increased osteoclast differentiation by *S*. *aureus* targeting osteoclast progenitors stimulated by endogenous RANKL produced in the periosteum/endosteum also may contribute to the enhanced bone resorption.

It has been reported that activation of TLR2 inhibits RANKL-induced osteoclast formation in BMM cultures [21,35,36]. We, therefore, wondered how *S*. *aureus* could increase osteoclastogenesis and bone resorption in the *ex vivo* parietal bone organ cultures. To investigate if osteoclast progenitors in parietal bones were different from those in bone marrow we next used cell cultures of periosteal/endosteal cells from parietal bones and mouse bone marrow cultures and compared the effect *S*. *aureus* on non-stimulated and RANKL stimulated cells, respectively. When *S*. *aureus* was added together with RANKL to mouse bone marrow macrophage cultures we could confirm observations made by others [21,35,36] showing that the bacterium can abolish osteoclast differentiation. In contrast, when *S*. *aureus* was added together with RANKL to periosteal/endosteal cell cultures, no inhibition of osteoclastogenesis was observed. Similar to this finding, *S*. *aureus* did not affect bone resorption in the parietal bones stimulated by exogenous RANKL. These findings show that osteoclast progenitors in bone marrow and at bone surfaces are fundamentally different in their response to *S*. *aureus* and explain why *S*. *aureus* can stimulate osteoclast formation in intact bones despite its inhibitory effect on RANKL-stimulated bone marrow macrophages. Previously, we have similarly shown that also vitamin A and LPS *P*. *gingivalis* stimulate bone resorption in parietal bones and increase formation of bone resorbing osteoclasts in periosteal/endosteal cell cultures, while also inhibiting RANKL-stimulated osteoclast formation in bone marrow macrophage cultures [52,61,62]. All together, these observations indicate that studies on osteoclast formation should not only be based upon osteoclastogenesis in bone marrow macrophages but also include experiments using osteoclast progenitors present at the surfaces of bone.

When *S*. *aureus* was added to periosteal/endosteal cell cultures not stimulated with RANKL, we observed increased formation of osteoclasts, similar to the observations in the calvarial bones. This response was associated with increased mRNA expression of the osteoclastic genes *Acp5* and *Ctsk* as well as with *Tnsf11* mRNA, indicating that increased number of mature osteoclasts was due to increased differentiation of osteoclast progenitor cells due to increased RANKL in osteoblasts which are abundant in these cultures.

The reason for the different responsiveness of osteoclast progenitors in bone marrow and periosteum/endosteum is not known but could be due to differences in differentiation stage and/or to the microenvironment. If bone marrow macrophages are primed with RANKL before subsequent stimulation by LPS *E*. *coli* or *S*. *aureus* cell wall peptidoglycan, in the absence of RANKL, formation of mature osteoclasts is induced [21]. Similarly, the synthetic TLR2 agonists Pam2 and Pam3 [17,21], and the periodontal pathogen *P*. *gingivalis* acting through TLR2 [54], stimulate osteoclast formation in RANKL-primed bone marrow macrophages. These findings indicate that the differences between bone marrow macrophages and periosteal/endosteal osteoclast progenitor responses to stimulatory ligands may depend on the differentiation level of osteoclast progenitors. When we added *S*. *aureus* to RANKL-primed bone marrow macrophages, the cells started to differentiate and became TRAP^+^ but the mononuclear cells did not fuse to mature osteoclasts. In contrast, addition of the two synthetic TLR2 agonists Pam2 and Pam3 stimulated formation of mature osteoclasts to the same degree as RANKL. Similar to *S*. *aureus*, LPS *P*. *gingivalis* and HKLM, two other TLR2 agonists induced differentiation of TRAP^+^ mononuclear cells but not formation of mature osteoclasts. Pam2 and Pam3 robustly upregulated osteoclastic and osteoclastogenic genes such as *Ctsk*, *Acp5*, *Calcr*, *c-Fos* and *Nfatc1*, a response also observed after treatment with *S*. *aureus* and LPS *P*. *gingivalis* but to a much lesser degree. We do not know if the reason why *S*. *aureus* was unable to induce mature osteoclast formation was due to the quantitative differences in gene expression, or if the TLR2 in the RANKL-primed osteoclast progenitors are not fully activated by the bacterial agonist, in contrast to the synthetic ligands. Interestingly, we have found that LPS *P*. *gingivalis*, HKLM, Pam2 and Pam3 stimulate bone resorption in *ex vivo* parietal bones and *Tnfsf11* mRNA expression in osteoblasts through TLR2 to the same degree [52], indicating that TLR2 in osteoblasts and RANKL-primed bone marrow macrophages are not entirely similar. The fact that multinucleated osteoclast formation was observed in RANKL-primed bone marrow macrophages treated with *P*. *gingivalis* [54], in contrast to our findings showing differentiation of mononuclear osteoclasts, may be due to that whole bacteria was used instead of the LPS preparation used by us.

In agreement with the well-known consequence of TLR activation, *S*. *aureus* stimulated the expression of several cytokines such as IL-1β, IL-11, IL-6, LIF, OSM and TNF-α. Since these cytokines are potent stimulators of RANKL expression, osteoclast formation and bone resorption [22, 63–65], we assessed if RANKL and osteoclastogenesis induced by *S*. *aureus* was secondary to induction of these cytokines. Using neutralizing antibodies, we show, however, that the effect of *S*. *aureus* on bone resorption and RANKL formation in parietal bones and isolated osteoblasts is not mediated by these cytokines. *S*. *aureus* also enhanced the mRNA expression in parietal bones and isolated osteoblasts of *Ptgs2*, a key enzyme in prostaglandin biosynthesis. Although inhibition of prostaglandin biosynthesis in the parietal bones and osteoblasts decreased *S*. *aureus* induced *Tnfsf11* mRNA expression, no effect on bone resorption was observed; most likely due to the robust stimulation of *Tnfsf11* still observed in *S*. *aureus* stimulated bones co-treated with the prostaglandin inhibitor. The reason why inhibition of prostaglandin biosynthesis abolished *S*. *aureus* induced *Tnsf11* mRNA expression in calvarial osteoblasts, while only partially decreased this response in calvarial bones, might be due to that *S*. *aureus* can induce RANKL in cells present in the calvarial bones, but not in the osteoblasts cultures, and that the calvarial cells are insensitive to prostaglandins. One such possibility is osteocytes which have been shown to be more important producers of RANKL than osteoblasts [26, 27].

*S*. *aureus* may not cause decreased bone mass only by increasing bone resorption but also by decreasing bone formation. Several studies using human and mouse osteoblasts have shown that *S*. *aureus* can inhibit expression of genes associated with osteoblast differentiation and bone formation [28–32]. We observed a similar effect using bone organ cultures in which *S*. *aureus* decreased the mRNA expression genes encoding osteocalcin, procollagen type I, alkaline phosphate and Runx2. Nfatc1 is most well known as a master regulator of osteoclast differentiation [46], but is also expressed in osteoblasts. The role of Nfatc1 in bone formation is controversial with both stimulatory [55, 56] and inhibitory [57, 58] effects observed. We found, however, that stimulation of Nfatc1 by *S*. *aureus* in the calvarial bones was not involved in the inhibition of the osteoblast anabolic genes.

Since the array of symptoms displayed by patients with *S*. *aureus* infection is correlated to the arsenal of virulence factors exhibited by *S*. *aureus*, we used two different strains of *S*. *aureus* and evaluated the role of toxins in *S*. *aureus* induced bone resorption. Our findings demonstrate that the ability of toxin production has no significant effect on bone resorption stimulated by *S*. *aureus* in isolated *in vitro* assays. Most likely, the toxin production characteristics of certain *S*. *aureus* strains have favorable effects on invasiveness, escape and damage of the immune system and exacerbating the infection and inflammation *in vivo*.

In summary, *S*. *aureus* targets osteoblasts (or maybe osteocytes) through TLR2 causing increased RANKL and periosteal/endosteal osteoclast formation and bone resorption with no signs of *S*. *aureus* targeting the subpopulation of osteoclast progenitors present at the surfaces of bone. The finding that activation of TLR2 in a subpopulation of osteoclast progenitors present in bone marrow which have been primed by RANKL results in osteoclast differentiation indicate the possibility that S. aureus might increase bone resorption also through activation of osteoclast progenitors at a certain differentiation level. The relative importance of osteoblasts/osteocytes and osteoclast progenitors for the bone resorptive effect by *S*. *aureus* has to be assessed in mice (and/or bone organ cultures) with cell specific deletion of TLR2.

## Supporting Information

S1 Fig *S*. *aureus* and *S*. *aureus Tox* time- and concentration-dependently increased the mRNA expression in parietal bones of *Csf1r* (A, B), *Csf1* (C, D) without affecting the mRNA expression of *Tnfrsf11a* (E, F) and *Tnfrsf11b* (G, H). In A, effects were statistically significant at 12 and 48 h (*P*<0.01) and at 24 h (*P*<0.001). In B, effects were statistically significant by 10^6^ and 3x10^7^ (*P*<0.01) and by 3x10^6^ and 10^7^ (*P*<0.001) CFU/ml. In C, effects at 1–48 h were statistically significant (*P*<0.001). In D, effects were statistically significant by 3x10^5^–10^7^ (*P*<0.001) and by 3x10^7^ (*P*<0.01) CFU/ml. No statistically effects were obtained in experiments shown in E-H.
(TIF)

S2 Fig Anti-IL-1β and anti-TNF-α effectively inhibit *Tnfsf11* mRNA in parietal bones induced by IL-1β and TNF-α, respectively (A), anti-IL-11, anti-LIF and anti-OSM effectively inhibit *Tnfsf11* mRNA induced by IL-11, LIF and OSM, respectively (B) and anti-IL-6 inhibit *Tnfsf11* mRNA induced by co-treatment with IL-6 and IL-6 soluble receptor (C). *P<0.05, **P<0.01 and ***P<0.001 compared to unstimulated control or to cytokine stimulated bones.
(TIF)

S3 Fig A-D show that *S*. *aureus* inhibits bone formation in organ cultured mouse parietal bones as assessed by decreased mRNA expressions of *Bglap* (A), *Akp1* (B), *Procol1a1* (C) and *Runx2* (D). In E-H is demonstrated that the osteoclast inhibitor OPG does not affect the inhibition of *Bglap* (E), *Akp1* (F), *Procol1a1* (G) and *Runx2* (H) induced by *S*. *aureus* in the parietal bones. *P<0.05, **P<0.01 and ***P<0.001 compared to unstimulated control bones.
(TIF)
