# Supplementary figures and images for: Toll-Like Receptor 2 Stimulation of Osteoblasts Mediates Staphylococcus Aureus Induced Bone Resorption and Osteoclastogenesis through Enhanced RANKL
Source: PLoS One. 2016 Jun 16;11(6):e0156708. doi: 10.1371/journal.pone.0156708 (PMC4911171; doi:10.1371/journal.pone.0156708)

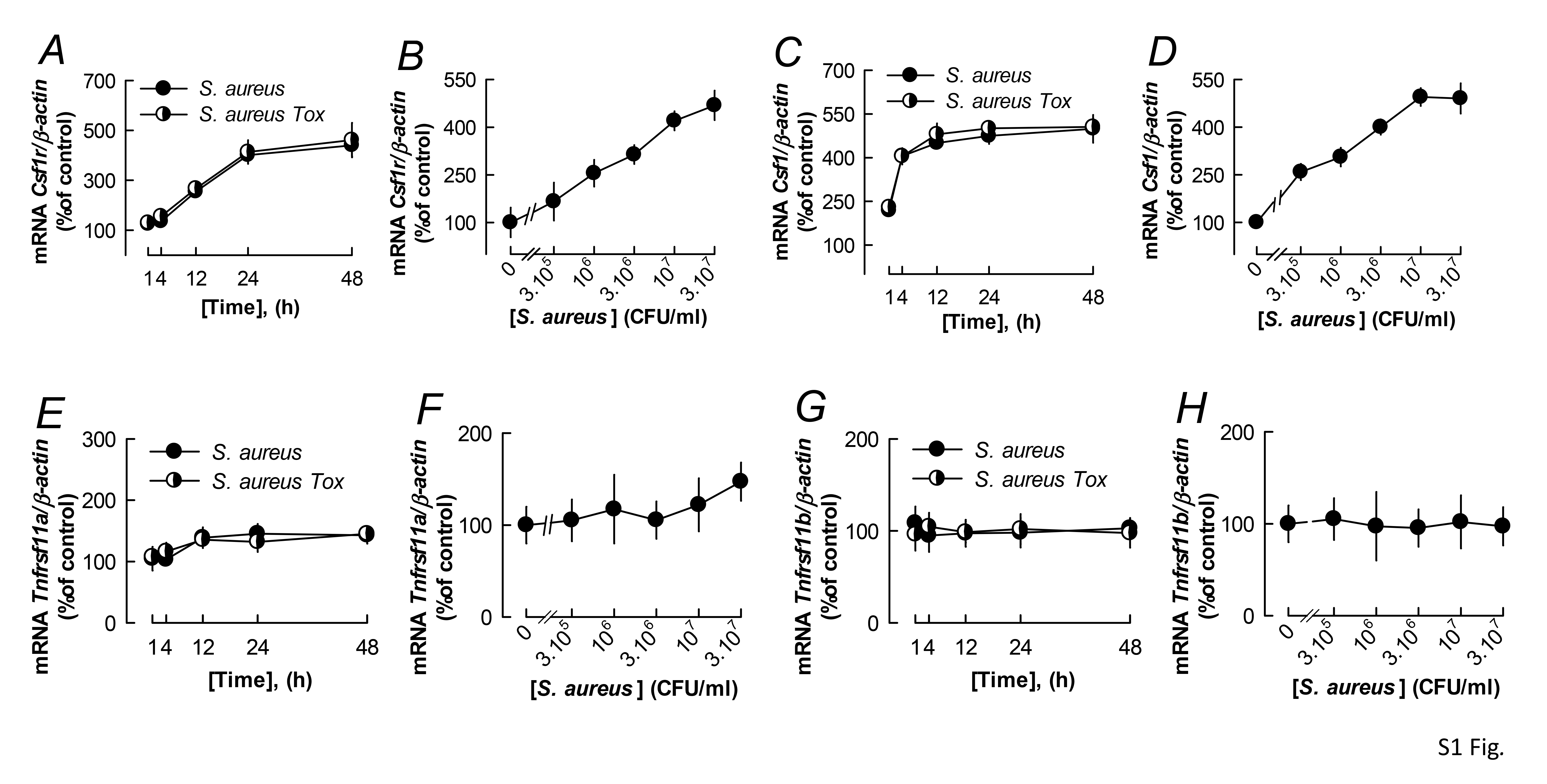

Supplement: S1 Fig — In A, effects were statistically significant at 12 and 48 h (P<0.01) and at 24 h (P<0.001). In B, effects were statistically significant by 106 and 3x10^7^ (P<0.01) and by 3x10^6^ and 107 (P<0.001) CFU/ml. In C, effects at 1–48 h were statistically significant (P<0.001). In D, effects were statistically significant by 3x10^5^–107 (P<0.001) and by 3x10^7^ (P<0.01) CFU/ml. No statistically effects were obtained in experiments shown in E-H. (TIF) [file pone.0156708.s001.tif]

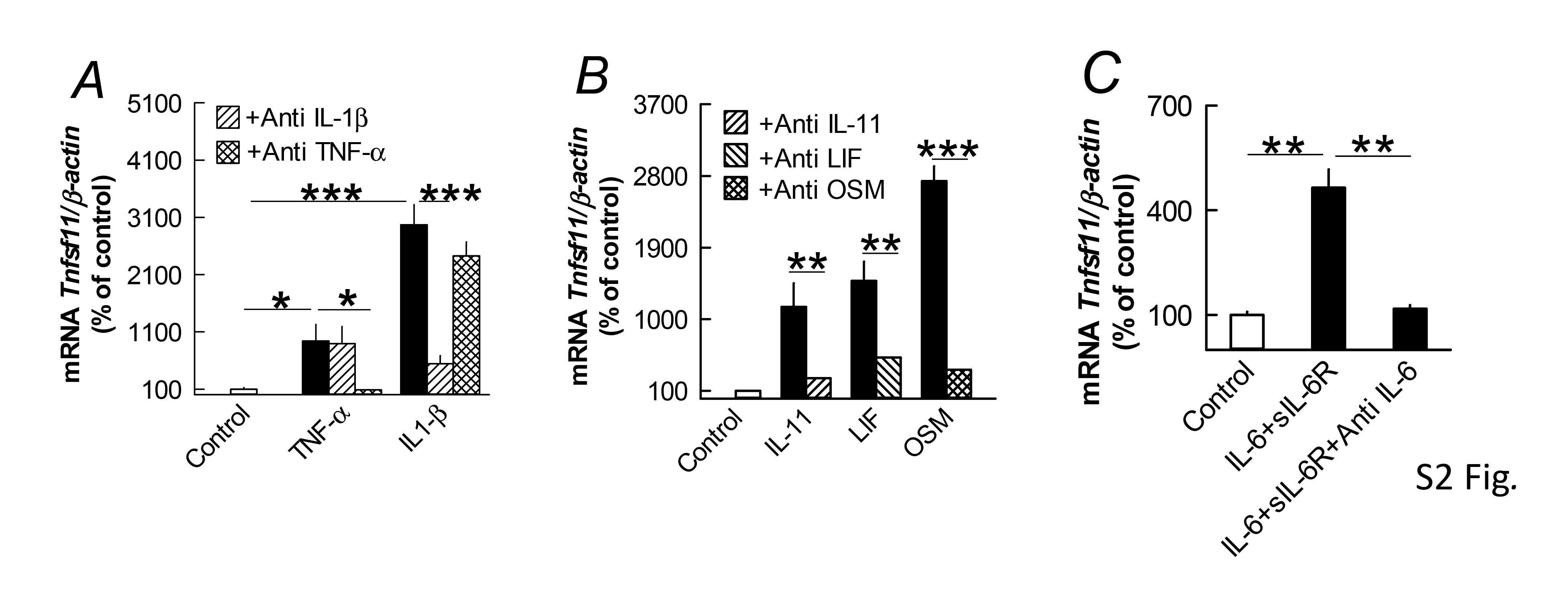

Supplement: S2 Fig — *P<0.05, **P<0.01 and ***P<0.001 compared to unstimulated control or to cytokine stimulated bones. (TIF) [file pone.0156708.s002.tif]

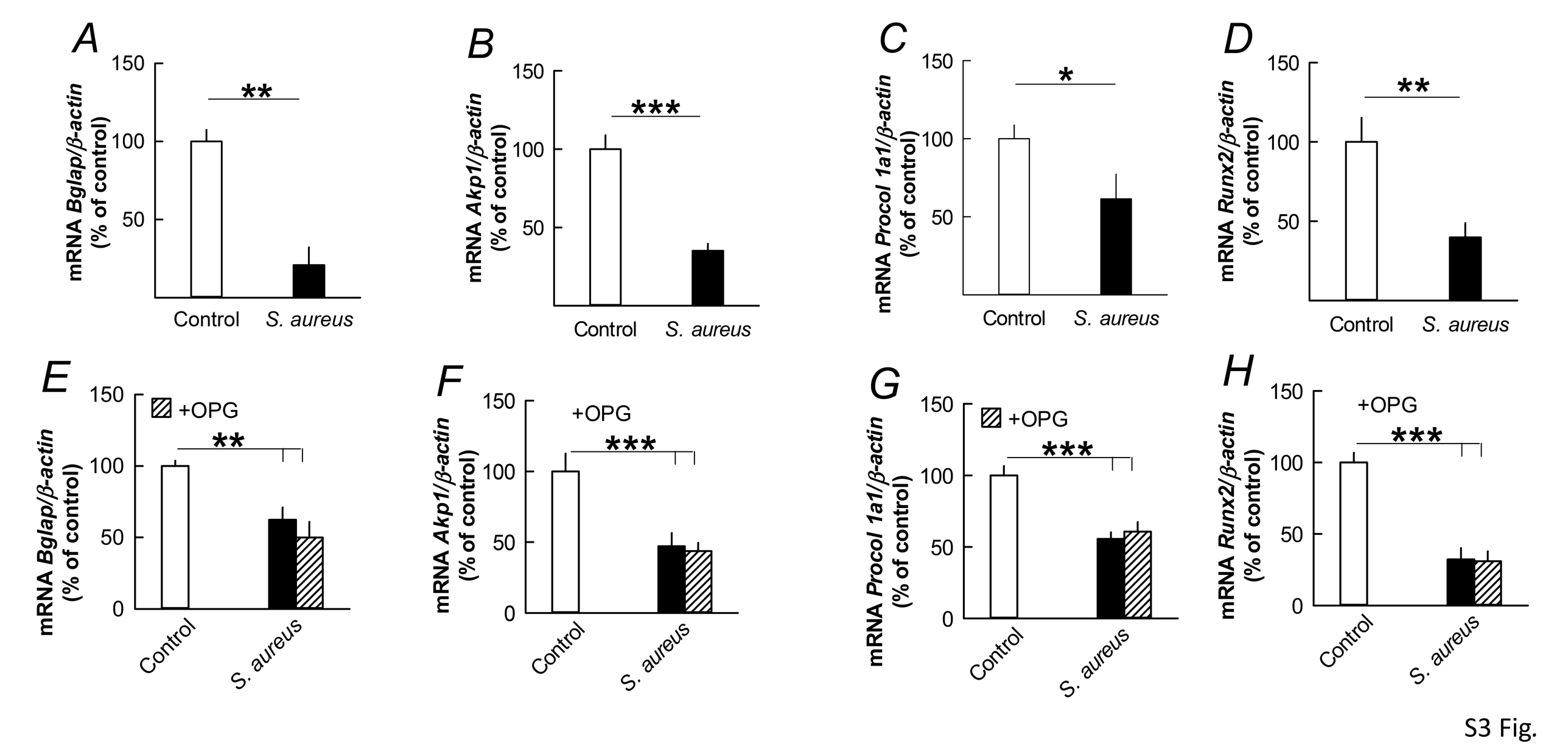

Supplement: S3 Fig — A-D show that S. aureus inhibits bone formation in organ cultured mouse parietal bones as assessed by decreased mRNA expressions of Bglap (A), Akp1 (B), Procol1a1 (C) and Runx2 (D). In E-H is demonstrated that the osteoclast inhibitor OPG does not affect the inhibition of Bglap (E), Akp1 (F), Procol1a1 (G) and Runx2 (H) induced by S. aureus in the parietal bones. *P<0.05, **P<0.01 and ***P<0.001 compared to unstimulated control bones. (TIF) [file pone.0156708.s003.tif]
